# Identifying avian malaria vectors: sampling methods influence outcomes

**DOI:** 10.1186/s13071-015-0969-0

**Published:** 2015-07-11

**Authors:** Jenny S. Carlson, Erika Walther, Rebecca TroutFryxell, Sarah Staley, Lisa A. Tell, Ravinder N. M. Sehgal, Christopher M. Barker, Anthony J. Cornel

**Affiliations:** Department of Entomology, University of California, Davis, CA USA; Department of Biology, San Francisco State University, San Francisco, CA USA; Department of Entomology and Plant Pathology, The University of Tennessee, Knoxville, TN USA; Department of Medicine and Epidemiology, University of California, Davis, CA USA; Department of Pathology, Microbiology, and Immunology, University of California, Davis, CA USA

**Keywords:** Avian malaria, *Culex* spp., *Plasmodium* spp., Vector competence, Vector compatibility filter, Trapping bias

## Abstract

**Background:**

The role of vectors in the transmission of avian malaria parasites is currently understudied. Many studies that investigate parasite-vector relationships use limited trapping techniques and/or identify potential competent vectors in the field in such ways that cannot distinguish between an infected or infectious vector. Without the use of multiple trapping techniques that address the specific biology of diverse mosquito species, and without looking at the infection status of individual mosquitoes, it is not possible to make dependable conclusions on the role of mosquitoes in the transmission of avian malaria parasites.

**Methods:**

We conducted two years of mosquito collections at a riparian preserve in California where a wide diversity of species were collected with multiple trap types. We hypothesized that competent mosquito species can influence the distribution and diversity of avian malaria parasites by acting as a compatibility filter for specific *Plasmodium* species. To determine the infection status of all individual mosquitoes for *Plasmodium* species/lineages, amplification within the cytochrome *b* gene was carried out on over 3000 individual mosquito thoraxes, and for those that tested positive we then repeated the same process for abdomens and salivary glands.

**Results:**

Our data show heterogeneity in the transmissibility of *Plasmodium* among ornithophillic mosquito species*.* More specifically, *Culex stigmatosoma* appears to not be a vector of *Plasmodium homopolare*, a parasite that is prevalent in the avian population, but is a vector of multiple other *Plasmodium* species/lineages.

**Conclusions:**

Our results suggest that conclusions made on the role of vectors from studies that do not use different mosquito trapping methods should be re-evaluated with caution, as we documented the potential for trapping biases, which may cause studies to miss important roles of specific mosquito species in the transmission of avian malaria. Moreover, we document heterogeneity in the transmission of *Plasmodium* spp. by mosquitoes can influence *Plasmodium* diversity and prevalence in specific locations to *Plasmodium-*vector incompatibilities.

## Background

Avian malaria is caused by infections with protozoan parasites in the genus *Plasmodium* [1], which are closely related to parasites in the genera *Haemoproteus* and *Leucocytozoon*. Currently, there are more than 50 described *Plasmodium* species that infect avian hosts [2–4]. Unlike the human malaria system, in which parasites are transmitted solely by mosquitoes in the subfamily Anophelinae, avian malaria is transmitted by a more diverse set of species within Anophelinae and Culicinae subfamilies [2, 5, 6].

It has been proposed that avian *Plasmodium* parasites shift hosts readily, which explains why there is poor matching between *Plasmodium* species/lineages and bird taxa in phylogenetic trees [7, 8]. Vertebrate host switching is enabled by the vector because birds become infected by infective mosquito bites. Insufficient blood feeding specialization by ornithophillic mosquitoes results in the distribution of *Plasmodium* species/lineages among a diverse array of avian hosts, reducing sympatric parasite assortative mating opportunities, and lessening conditions conducive for specific parasite co-evolutionary processes to occur [7, 9, 10]. However, the role of vectors in the transmission of avian malaria parasites is currently underestimated, and it is not clear how important vectors are in the structuring of *Plasmodium*-host relationships.

Multiple studies have been conducted to evaluate the role of mosquitoes in avian malaria transmission [3, 9–21]. Although these studies are informative, there is a need for more standardized methods to determine the relative contributions of the various mosquito species to biogeographical structuring of *Plasmodium* populations. Testing of whole mosquitoes only provides partial information because it establishes whether mosquitoes are infected, but not whether parasites are transmissible [22]. The contribution of each species as a vector can be better determined by showing the presence of sporozoites in the salivary glands in field-collected specimens; however, not all vectors are capable of transmitting sporozoites by a bite, even when they are present in the salivary glands [23]. Detection of sporozoites from the salivary glands, therefore, show that sporogony can be completed, but it should be followed up by studies that include vector competence assays and field observations, such as host feeding propensities, vector and parasite densities, seasonality, and longevity to determine each species’ relative importance as a vector.

In an attempt to address whether or not mosquitoes play a role in the structuring of *Plasmodium*-host relationships, we undertook a 2-year study in central California where the biology of most California mosquito species is well known. In this study, mosquitoes were not pooled and the salivary glands were tested individually. Moreover, we attempted to collect a wide diversity of mosquito species by using multiple trap types. Traps vary in their attractiveness depending on mosquito species and gonotrophic condition, and studies based on one trap type could miss important information about the role of vectors, or not identify a competent vector altogether at a specific location [24–28]. To connect the community structure of *Plasmodium* in mosquitoes to that in birds*,* concurrent detections were also made from birds in the same area “Walther et al., unpublished observations.” We hypothesized that vector mosquito species can influence the distribution and diversity of avian malaria parasites by acting as a compatibility filter for specific parasite species. To test this hypothesis we carried out the following aims: (1) to estimate the relative transmission potential of competent vectors in the field; and (2) to determine if *Plasmodium-*vector relationships exist to a degree where specific incompatibilities occur between *Plasmodium* species/strains and mosquito species.

## Methods

### Mosquito collection

Mosquitoes were collected in 2011 and 2012 at China Creek Park in Fresno County, California (36°44' N, 119°29' W, 120 m asl). China Creek Park is a riparian 1.2 km^2^ park including two small ponds and a creek. This park was chosen for its species richness in both avian and mosquito populations. Mosquitoes have opportunities to feed on multiple host types at China Creek Park, including a wide range of bird species, rodents, lagomorphs, and larger mammals such as coyotes and bobcats. Numerous properties surrounding China Creek Park are used for cattle grazing and one property owner grazes cattle within the park boundary.

In 2011, collections were carried out daily between May 27^th^ to June 3^rd^, June 20^th^ to June 30^th^, and September 31^st^ to October 2^nd^. In 2012, daily collecting dates were March 17^th^, June 22^nd^ to June 30^th^, and October 6^th^ to October 10^th^. Additionally, overwintering mosquitoes were collected on January 20^th^, 2013 using a backpack aspirator (BioQuip, Rancho Dominguez, CA).

Mosquitoes were collected in 2011 with gravid traps containing grass-infused water serving as the attractant [29]; Encephalitis Virus Surveillance (EVS) traps baited with dry ice as the source of CO_2_ [30]; standing red boxes [31], which provided a shady environment favorable for resting mosquitoes; net traps [3] baited with alternating dry ice, canaries, and pigeons placed in bird cages as the sources of CO_2_ and other attractants (e.g., heat, droppings, etc.); and Ehrenberg pigeon traps baited with restrained pigeons as described by Downing and Crans [32] to attract ornithophillic mosquitoes. The use of birds as an attractant in this study complies with IACUC permit 16440 UC Davis. In 2012, only gravid traps, EVS traps and red boxes were used; we eliminated nets and Ehrenberg traps due to a very low prevalence of *Plasmodium* infections among the mosquitoes collected in 2011.

Gravid traps, EVS traps, and agricultural nets were deployed at dusk and left running for 12 h. Ehrenberg traps were operated for 4 h after sunset. Red boxes were inspected for resting mosquitoes at dusk and at dawn. Collected mosquitoes were brought back to the laboratory for identification and dissection. All mosquitoes were first anesthetized with triethylamine (Sigma-Aldrich, St. Louis, MO), then species were identified using dichotomous keys by Darsie and Ward [33] and by Bohart and Washino [34]. Once identified, salivary glands, the abdomen, and the thorax were dissected from each mosquito and placed in individual 95 % ethanol vials (i.e., three vials per mosquito). To prevent contamination between body parts and between mosquitoes, forceps were first dipped in 95 % ethanol and wiped dry with Kimwipes (Kimtech, Roswell, GA) after a brief immersion in 100 % bleach. All tubes were stored in a walk in refrigerator (4 °C) for no longer than one month until DNA extractions were performed.

### Parasite screening in mosquitoes

Thoraxes of each individual mosquito were homogenized using the Qiagen stainless steel 5 mm beads and the Qiagen TissueLyser (Qiagen, Valencia, CA) for 3 min at the maximum speed before the overnight incubation in the lysis buffer provided in the kit. DNA was then extracted from thoraxes following the Qiagen BioSprint 96 DNA Tissue Extraction Kit (Qiagen, Valencia, CA) protocol and using the BioSprint 96 instrument (Qiagen, Valencia, CA).

Extracted DNA from mosquito thoraxes was screened for both *Plasmodium* and *Haemoproteus* parasite DNA using polymerase chain reactions (PCR). We amplified 478 bp (excluding primers) of the parasite mitochondrial cytochrome oxidase subunit-*b* gene (cyt *b*) using nested primers HaemNF, HaemNR2, HaemF and HaemR2 as described in Waldenström et al. [35]. PCRs were carried out in a 20 μl reaction mixture using AccuPower Taq PCR PreMix (Bioneer Corporation, Daejeon, Republic of Korea) containing 1 μl of each 10 μM primer, 0.5 μl of Bovine Serum Albumin (BSA), 2 μl of template DNA and 15.5 μl of purified water. All PCR products were viewed on 1.8 % agarose gels stained with ethidium bromide. Positive PCR products were then bi-directionally sequenced by Elim Biopharmaceuticals Inc., Hayward, CA. All parasite lineages sequenced were aligned and edited using Sequencher 4.8 (GeneCodes, Ann Arbor, MI). The sequences obtained from the mosquitoes were aligned and compared to sequences in the NCBI nucleotide database using the BLAST® search option to identify to genus and species/lineage. Parasite sequences that differed by 1–3 bp were considered distinct lineages [36], after repeating an independent PCR and sequencing for verification. Sequence chromatograms were visually inspected for double peaks, which would indicate the presence of a multiple infection. All final sequences were deposited to GenBank [GenBank:KJ620777,KJ620779,KJ620781,KJ620783,KJ620784,KJ620788-KJ620792,KJ482708] and MalAvi [37].

The process described above was then repeated for the corresponding abdomen and salivary glands of all thorax positive individuals. Mosquitoes that were thorax and abdomen positive were considered infected (obtained the parasite with bloodmeal)- to be very conservative, whereas mosquitoes that were salivary gland positive were considered more likely to be infective (capable of transmitting the parasite with the next bloodmeal).

### Identifying species in the Culex pipiens complex

To distinguish between members belonging to the *Cx. pipiens* complex, PCR was conducted with mosquito DNA extracted from thoraxes to amplify sections of exons 2 and 3 and the entire intron II in the *ace*-2 gene. Diagnostic primers ACEpip and ACEquin (forward primers), along with B1246s (reverse primer), were used in the assay as described by Smith and Fonseca [38].

### Data analysis

*Plasmodium* and *Haemoproteus* mosquito thorax prevalence was calculated based on testing thoraxes for each lineage (% of infected individuals of the total collection for each lineage). The prevalence data is followed by a 95 % confidence interval. A Generalized Linear Model (GLM) was conducted to test the effects of (i) trap type on *Plasmodium* prevalence after adjusting for the difference among mosquito species, and (ii) mosquito species on *Plasmodium* prevalence after adjusting for trap types. Only EVS and gravid traps were included in the analysis because they were the only two that provided an approximately consistent sampling effort. Count data could not be compared among trap types due to differences in the trapping effort over time, particularly because some traps were only used during the first year of sampling. However, to graphically represent the degree of association between the response categories of trap type and mosquito species collected, we performed a Correspondence Analysis (CA), which makes no assumption about distributions [39]. All analyses were conducted in R [40].

### Phylogenetic analysis

*Plasmodium* lineages from mosquitoes were combined in the same file with those from avian hosts at China Creek Park “Walther et al., unpublished observations” and aligned in Sequencher 4.8 (GeneCodes, Ann Arbor, MI) to determine which sequences infected both mosquito and avian hosts (defined by a 100 % sequence match). Lineages were given names in accordance to the proposed lineage naming criteria discussed by participants at the 2013 Malaria and Related Haemospordian Parasites of Wildlife Research Coordination Network meeting, Vilnius, Lithuania. Lineages are to reflect a code for the host (from which the parasite was first obtained), the locality code, the initials of the person that collected the sample, and a unique assigned code for the parasite lineage that would also reflect to which genus the parasite belongs to (i.e., P for *Plasmodium*).

**Fig. 1:**
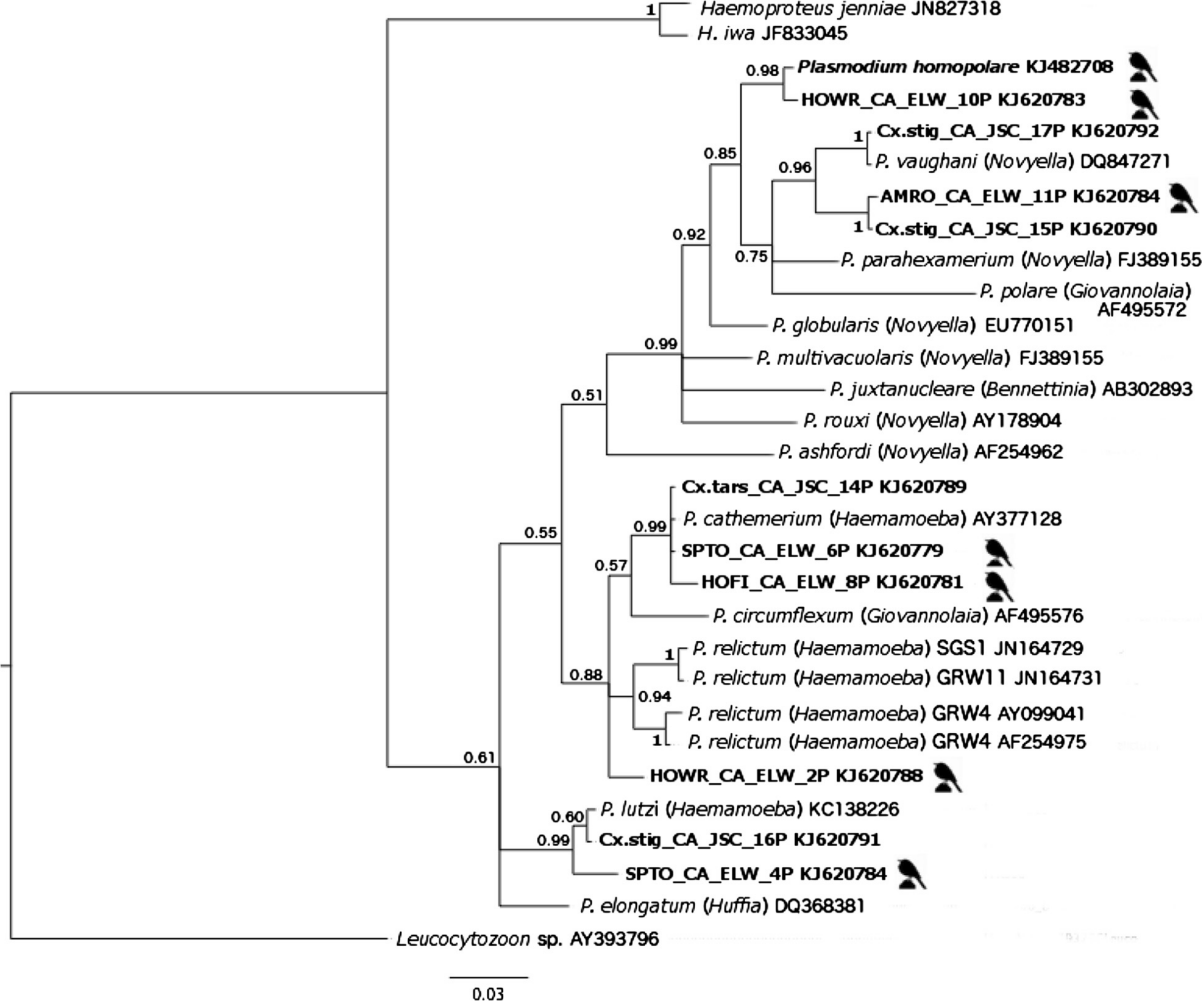
Bayesian phylogeny of 27 mitochondrial cytochrome *b Plasmodium* spp./lineages found in mosquitoes and birds, along with two *Haemoproteus spp.* A *Leucocytozoon* spp. was used as the out-group. Numbers at each node represent the Bayesian posterior probabilities. Lineage names in bold (total of 11) were obtained from China Creek Park. Lineages followed by a bird symbol indicates that it was also detected in birds trapped at China Creek Park. All lineages used for this analysis are delineated by the parasite name followed by the Genbank accession number, and for *Plasmodium* spp. the subgenus is also provided in parenthesis

Phylogenetic relationships were analyzed using the best fit GTR + G + I model of molecular evolution as calculated with MrModeltest [41]. A Bayesian phylogenetic tree was constructed in MrBayes version 3.1.2 [42]. Twenty-seven *Plasmodium* lineages were used for the phylogenetic analysis of which 11 lineages were obtained from our study, and we included sequences previously reported in birds of 16 *Plasmodium* spp. and 2 *Haemoproteus* spp., while *Leucocytozoon* sp. was designated as the outgroup sequence (see Fig. 1 for accession numbers).

Two Markov Chain Monte Carlo (MCMC) simulations were run simultaneously for 10 million generations with sampling every 200 generations. This resulted in a total of 100,000 trees, of which the first 25,000 trees (12,500 from each MCMC simulation) were discarded from the sample as the “burn-in” period. The remaining trees were used to construct a majority rule consensus tree and to calculate the posterior probabilities of the individual clades.

## Results

### Mosquito collections

A total of 3083 mosquitoes representing 15 species were collected in 2011 (2063) and in 2012 (1020) (Table 1). Species in the genus *Culex* represented 72 % (2231 of 3083) of the collection and included 6 different species. Of the *Culex* species collected 693 were *Cx. stigmatosoma* (Dyar 1907), 551 were identified as belonging to the *Cx. pipiens* complex, 464 were *Cx. tarsalis* (Coquillett 1896), 297 were *Cx. erythrothorax* (Dyar 1907), 166 were *Cx. restuans* (Theobald 1901), and 59 were *Cx. thriambus* (Dyar 1921). In California, *Cx. restuans* has distinctive tarsal white bands, which makes it easily distinguishable from members of the *Cx. pipiens* complex [34]*.* A total of 337 *Aedes* species were collected, of which 324 were *Ae. vexans* (Meigen 1830), 9 were *Ae. washinoi* (Lanzaro and Eldridge 1992) and 4 were *Ae. sierrensis* (Ludlow 1905)*.* Two *Anopheles* species were collected, of which, 168 were *An. punctipennis* (Say 1823) and 88 were *An. freeborni* (Aitken 1939)*.* Lastly, 4 species of *Culiseta* were represented in the collection; 240 were *Cs. particeps* (Adams 1903), 8 were *Cs. inornata* (Williston 1893)*,* 7 were *Cs. incidens* (Thomson 1869)*,* and 4 were *Cs. impatiens* (Walker 1848)*.*

**Table 1:** Total number of collected and of *Plasmodium* and *Haemoproteus* positive mosquitoes per trap type

		Collecting Year 2011						Collecting Year 2012			
		Totals by trap type						Totals by trap type			
Mosquito Species (Species codes for CA)		EVS	Gravid	RB	Net	E	N	EVS	Gravid	RB	N
*Cx. stigmatosoma*											
	Total trapped	12	326	0	4	31	**373**	12	306	0	**318**
(Cx_stig)											
	Total of positives	0	27, 1^a^	0	0	1	**28, 1** ^a^	1	24	0	**25**
*Cx. tarsalis*											
	Total trapped	207	1	15	31	62	**316**	143	0	3	**146**
(Cx_tar)											
	Total of positives	7	0	1	1, 1^a^	2^a^	**9, 3** ^a^	6	0	0	**6**
*Cx. restuans*											
	Total trapped	7	97	0	9	10	**123**	1	42	0	**43**
(Cx_res)											
	Total of positives	1^a^	1	0	0	1	**2, 1** ^a^	0	1	0	**1**
*Cx. pipiens*											
	Total trapped	110	103	3	43	4	**263**	61	227	0	**288**
(Cx_pip)											
	Total of positives	0	0	0	1^a^	0	**1** ^a^	0	3	0	**3**
*Cx. erythrothorax*		105	18	2	56	8	**189**	106	2	0	**108**
	Total trapped										
(Cx_ery)											
	Total of positives	0	0	0	0	0	**0**	0	0	0	**0**
*Cx. thriambus*											
	Total trapped	0	12	0	0	3	**15**	6	38	0	**44**
(Cx_thr)											
	Total of positives	0	0	0	0	0	**0**	0	0	0	**0**
*Cs. impatiens*											
	Total trapped	0	2	0	0	1	**3**	1	0	0	**1**
(Cs_imp)											
	Total of positives	0	0	0	0	0	**0**	0	0	0	**0**
*Cs. incidens*											
	Total trapped	1	0	0	1	0	**2**	2	2	1	**5**
(Cs_inc)											
	Total of positives	0	0	0	0	0	**0**	0	0	0	**0**
*Cs. inornata*											
	Total trapped	1	1	0	4	0	**6**	2	0	0	**2**
(Cs_ino)											
	Total of positives	0	0	0	0	0	**0**	0	0	0	**0**
*Cs. particeps*											
	Total trapped	61	17	38	113	1	**230**	9	0	1	**10**
(Cs_par)											
	Total of positives	1, 2^a^	0	1	0	0	**2, 2** ^a^	0	0	0	**0**
*Ae. sierrensis*											
	Total trapped	3	0	1	0	0	**4**	0	0	0	**0**
(Ae_sie)											
	Total of positives	0	0	0	0	0	**0**	0	0	0	**0**
*Ae. vexans*											
	Total trapped	42	0	0	274	0	**316**	7	1	0	**8**
(Ae_vex)											
	Total of positives	1^a^	0	0	3^a^	0	**4** ^a^	0	0	0	**0**
*Ae. washinoi*											
	Total trapped	0	0	4	4	0	**8**	1	0	0	**1**
(Ae_was)											
	Total of positives	0	0	0	0	0	**0**	0	0	0	**0**
*An. freeborni*											
	Total trapped	3	0	36	28	0	**67**	9	3	9	**21**
(An_fre)											
	Total of positives	1^a^	0	0	0	0	**1** ^a^	0	0	0	**0**
*An. punctipennis*											
	Total trapped	8	0	30	110	0	**148**	10	0	8	**18**
(An_pun)											
	Total of positives	0	0	0	1^a^	0	**1** ^a^	0	0	0	**0**
Totals for all species											
	Total trapped	560	577	129	677	120	**2063**	370	621	22	**1013**
	Total of positives	8, 5^a^	28, 1^a^	2	1, 6^a^	2, 2^a^	**41, 14** ^a^	7	28	0	**35**

Numbers of each mosquito species collected and total of individuals positive in their thoraxes for *Plasmodium* spp. and for *Haemoproteus* spp. (indicated by superscripted ‘a’) are provided for each trap type. In the column that lists each mosquito species also provides the abbreviation that is used for the Correspondence Analysis (CA) in Fig. 3. Numbers collected and total of positive individuals are reported for 2011 and 2012 (bolded N = overall number of individuals per species collected in all trap types and overall number of positive individuals per collecting year).

The abbreviations for each trap type are as follows:

*EVS* encephalitis virus surveillance traps baited with CO_2_, *G* gravid traps used with grass infused water as an attractant, *RB* red boxes serving as a resting box, *Net* agricultural net containing coolers with dry ice as a source of CO2 or birds, *E* ehrenberg trap baited with a pigeon

**Fig. 2:**
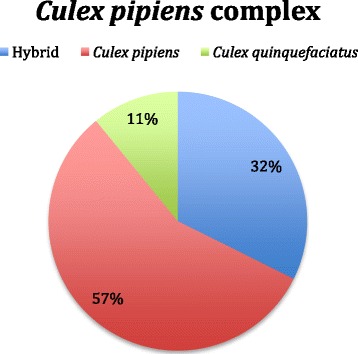
Identification of members of the *Culex pipiens* complex. A total of 504 individuals identified as members of the *Culex pipiens* complex were screened for species identification by amplifying part of the *ace*-2 gene following the protocol described by Smith and Fonseca [33]. Of the 504, 32 % (163/504) were identified as *Culex pipiens-Culex quinquefaciatus* hybrids, 57 % (286/504) were identified as *Culex pipiens*, and 11 % (55/504) were identified as *Culex quinquefasciatus*

The Smith and Fonseca assay [38] distinguished between 504 of the 551 collected members of the *Cx. pipiens* complex. A total of 47 amplifications failed due to the degradation of the extracted DNA. Figure 2 depicts the percentages of *Cx. pipiens* (Linnaeus 1758), *Cx. quinquefasciatus* (Say 1823), and their hybrids.

In 2011, all five trap types were used (EVS, gravid, nets, Ehrenberg traps and red boxes), but in 2012 only three of the five were used (EVS, gravid, and red boxes) because of time and resource constraints, and because the other two trapping methods yielded few infected mosquitoes in 2011. The predominant mosquito collected in EVS traps was *Cx. tarsalis*, 207 in 2011 and 143 in 2012. Gravid traps attracted mostly Cx*. stigmatosoma*, 326 in 2011 and 306 in 2012. Only one *Cx. tarsalis* was collected in a gravid trap, and only 24 Cx*. stigmatosoma* were collected in EVS traps over both years. *Cs. particeps* (38 in 2011 and one in 2012) and *An. freeborni* (36 in 2011 and 9 in 2012) were the most common species collected in red boxes. In 2011, *Ae. vexans* (274) and *Cx. tarsalis* (62) were the most commonly collected species in the net traps and in the Ehrenberg traps respectively.

It was not possible to conduct any test of independence on trap types and mosquito species due to unequal samples sizes and differences in the timing of operation of the various trap types. However, the GLM’ analysis on the prevalence of *Plasmodium* resulted in a slightly higher prevalence in gravid traps compared to EVS traps after adjustment for differences among species, but the difference was not significant (*P* = 0.22). *Plasmodium* prevalence in *Cx. tarsalis* was not significantly different from that in *Cx. stigmatosoma* (*P* = 0.75) after adjustment for trap type, but the prevalence was significantly lower in *Cx. pipiens* complex (*P* < 0.001) and *Cx. restuans* (*P* = 0.01) compared to *Cx. stigmatosoma*.

**Fig. 3:**
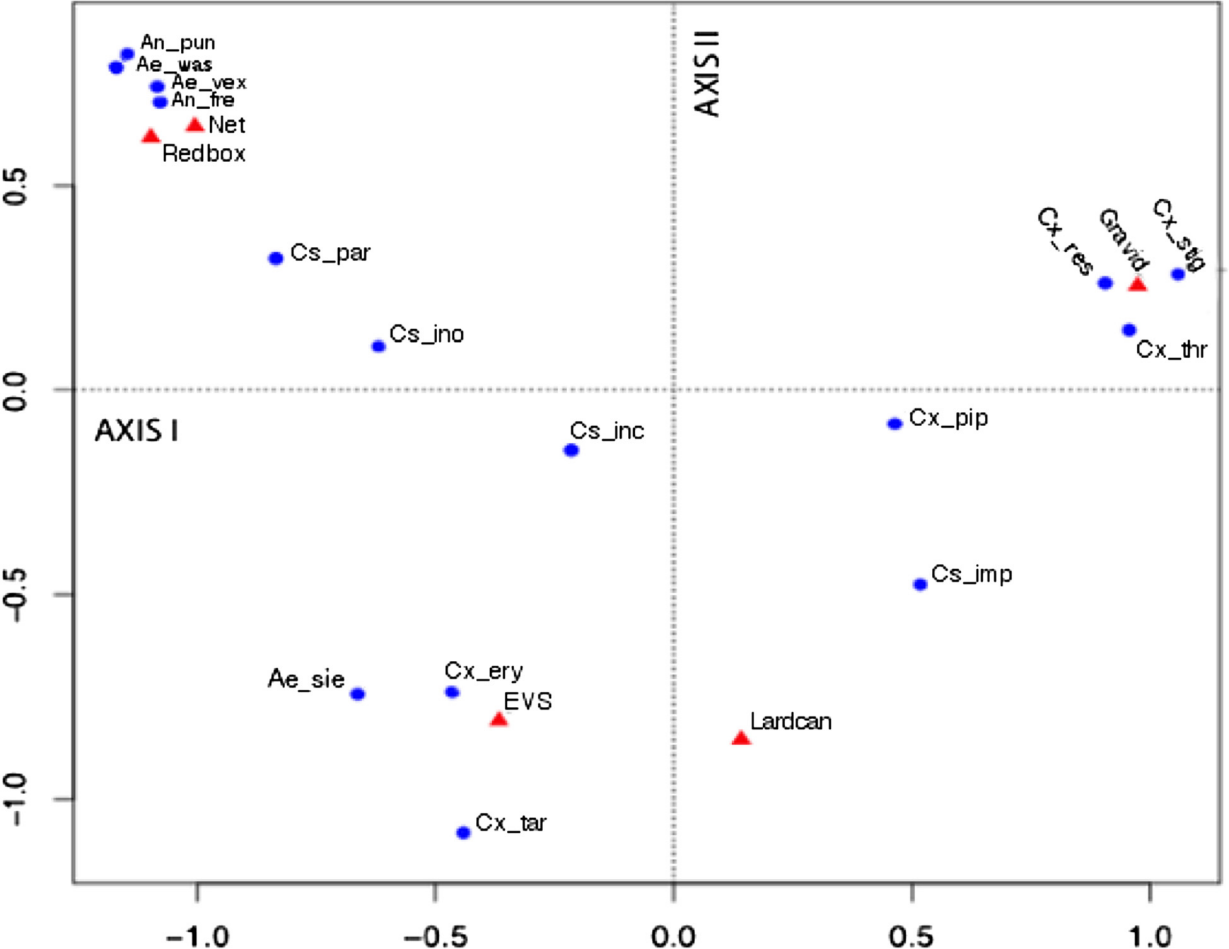
Correspondence analysis map of trap type and mosquito species variables. Circles correspond to mosquito species (see Table 1 for species code); triangles correspond to trap type

To graphically represent associations between mosquito species and trap types, a CA was conducted (Fig. 3) and showed three major associations. Net traps and the red boxes were strongly associated with collections of *An. punctipennis*, *An. freeborni*, *Ae. washinoi*, and *Ae. vexans*. EVS traps were strongly associated with collections of *Ae. sierrensis*, *Cx. erythrothorax* and *Cx. tarsalis.* Lastly, the gravid trap type was strongly associated with collecting *Cx. stigmatosoma*, *Cx. restuans*, and *Cx. thriambus*. Sample sizes were too small to make conclusions about the efficiency of the Ehrenberg trap type in collecting specific mosquito species.

### Vector-parasite associations

The overall *Plasmodium* prevalence in mosquito thoraxes was 2.5 %; 76 positive out of 3083 tested in both years combined (Table 2). No significant differences were observed in *Plasmodium* prevalence in mosquitoes between years 2011 and 2012, so henceforth we refer to infection and infective rates using the combined data set for the two years unless otherwise stated. The highest *Plasmodium* prevalence was found in *Cx. stigmatosoma.* Of the infected mosquitoes, 67 % were *Cx. stigmatosoma* in 2011 (28/42) and 74 % in 2012 (25/34). *Cx. tarsalis* was the second most frequently infected species with *Plasmodium* at 21 % in 2011 (9/42) and 18 % in 2012 (6/34). The remaining mosquito species infected with *Plasmodium* were *Cx. restuans,* members of the *Cx. pipiens* complex*,* and *Cs. particeps.*

**Table 2:** *Plasmodium* and *Haemoproteus* lineages detected in thoraxes for each mosquito species

Lineage	Genbank ID	N	% Positive (95 % credible interval)	Mosquito species							
				*Cx. pipiens*	*Cx. tarsalis*	*Cx. stigmatosoma*	*Cx. restuans*	*Cs. particeps*	*Ae. vexans*	*An. freeborni*	*An. punctipennis*
HOWR_CA_ELW_10P	*Plasmodium*	12	0.39 (0.22–0.68)	2	3	7	0	0	0	0	0
HOFI_CA_ELW_8	*Plasmodium*	13	0.42 (0.25–0.72)	0	4	9	0	0	0	0	0
HOWR_CA_ELW_2P	*Plasmodium*	9	0.29 (0.16–0.55)	1	2	6	0	0	0	0	0
Cx.stig_CA_JSC_17P	*Plasmodium*	7	0.23 (0.11–0.47)	0	0	7	0	0	0	0	0
Cx.stig_CA_JSC_16P	*Plasmodium*	2	0.06 (0.02–0.23)	0	0	2	0	0	0	0	0
Cx.stig_CA_JSC_15P	*Plasmodium*	7	0.23 (0.11–0.47)	0	1	5	1	0	0	0	0
SOSP_CA3P	*Plasmodium*	3	0.06 (0.04–0.28)	0	1	0	1	1	0	0	0
SPTO_CA_ELW_6P	*Plasmodium*	20	0.65 (0.42–1)	0	3	15	1	1	0	0	0
Cx.tars_CA_JSC_14P	*Plasmodium*	1	0.03 (0.01–0.18)	0	1	0	0	0	0	0	0
SPTO_CA_ELW_4P	*Plasmodium*	1	0.03 (0.01–0.18)	0	0	1	0	0	0	0	0
AMRO_CA_ELW_11P	*Plasmodium*	1	0.03 (0.01–0.18)	0	0	1	0	0	0	0	0
An.punc_CA_JSC_1H	*Haemoproteus*	11		1	2	1	1	2	3	0	1
Cx.tars_CA_JSC_2H	*Haemoproteus*	1		0	1	0	0	0	0	0	0
An.free_CA_JSC_3H	*Haemoproteus*	1		0	0	0	0	0	0	1	0
Ae.vexa_CA_JSC_4H	*Haemoproteus*	1		0	0	0	0	0	1	0	0
	Total	90		4	18	54	4	4	4	1	1

Confidence intervals are provided for the 11 *Plasmodium* lineages only (mosquitoes do not vector *Haemaproteus* species)

Of the 11 *Plasmodium* lineages detected in mosquito thoraxes, lineage SPTO_CA_ELW_6P [Genbank:KJ620779] was the most common (20/76 detections, 0.65 % prevalence; credible interval of 0.42–1 %), (Table 2). Lineage SPTO_CA_ELW_6P was detected primarily from *Cx. stigmatosoma* (15/20 infected), along with three detections from *Cx. tarsalis,* one from *Cx. restuans*, and one from *Cs. particeps. Cx. stigmatosoma* was also most frequently infected with all other lineages except for lineages SOSP_CA3P [Genbank:KJ482708] and Cx.tars_CA_JSC_14P [Genbank:KJ620789]. Lineage Cx.tars_CA_JSC_14P was detected in one *Cx. tarsalis,* and lineage SOSP_CA3P was detected in one *Cx. tarsalis,* one in *Cx. restuans* and one in *Cs. particeps.* Interestingly, members of *Cx. pipiens* complex were rarely infected, as only three of 551 were positive (prevalence of 0.005 %), which all three were identified as *Cx. pipiens-quinquefaciatus* hybrids.

Four *Haemoproteus* spp. were also detected from the thorax of 14 mosquitoes representing several species. Of the four species of *Haemoproteus,* lineage An.punc_CA_JSC_1H (Table 2) was the most common (11/14 infected) and was detected in many mosquito species, including *Ae. vexans, An. punctipennis, Cx. pipiens*, *Cx. restuans*, *Cx. stigmatosoma, Cx. tarsalis,* and *Cs. particeps.* The remaining three *Haemoproteus* lineages were detected from three different mosquitoes species (Table 2).

### Infection vs infectivity of different mosquito species

To distinguish between infected and infective status for *Plasmodium*, we tested the salivary glands and abdomens from the 76 thorax-positive mosquitoes. Table 3 lists all *Plasmodium-*positive body parts for each species tested. A total of 57 salivary glands of the 76 *Plasmodium* positive thoraxes were positive (75 %); however, the salivary glands of only three mosquito species were in fact positive (3/15 = 20 % of species). Salivary glands of *Cx. stigmatosoma* were positive for all lineages except SOSP_CA3P and Cx.tars_CA_JSC_14P, glands of *Cx. tarsalis* were positive for lineages HOWR_CA_ELW_10P, HOFI_CA_ELW_8P, HOWR_CA_ELW_2P, Cx.stig_CA_JSC_15P, SOSP_CA3P, and SPTO_CA_ELW_6P, and *Cx. restuans* salivary glands were positive for Cx.stig_CA_JSC_15P and SPTO_CA_ELW_6P.

**Table 3:** Body parts that tested positive for *Haemoproteus* spp. (thoraxes only) and for *Plasmodium* spp. (thoraxes, salivary glands, and abdomens) followed by the total prevalence for each body part

Mosquito species	Total collected	Total *Haemoproteus* positive thoraxes	*Haemoproteus* prevalence	Total *Plasmodium* positive thoraxes	*Plasmodium* positive thoraxes prevalence	Total *Plasmodium* positive salivary glands	*Plasmodium* positive salivary glands Prevalence	Total *Plasmodium* positive abdomens	*Plasmodium* positive abdomens prevalence
*Ae. sierrensis*	4	0	0	0	0	-	-	-	-
*Ae. vexans*	324	4	1.2 %	0	0	0	0	0	0
*Ae. washinoi*	9	0	0	0	0	-	-	-	-
*An. freeborni*	88	1	1.1 %	0	0	0	0	0	0
*An. punctipennis*	168	1	0.6 %	0	0	0	0	0	0
*Cx. erythrothorax*	297	0	0	0	0	-	-	-	-
*Cx. pipiens* complex	551	1	0.2 %	3	0.5 %	0	0	3	0.5 %
*Cx. restuans*	167	1	0.6 %	3	1.8 %	2	1.2 %	1	0.6 %
*Cx. stigmatosoma*	693	1	0.1 %	53	7.6 %	44	6.3 %	39	5.6 %
*Cx. tarsalis*	464	3	0.6 %	15	3.2 %	11	2.4 %	13	2.8 %
*Cx. thriambus*	59	0	0	0	0	-	-	-	-
*Cs. impatiens*	4	0	0	0	0	-	-	-	-
*Cs. incidens*	7	0	0	0	0	-	-	-	-
*Cs. inornata*	8	0	0	0	0	-	-	-	-
*Cs. particeps*	240	2	0.8 %	2	0.8 %	0	0	0	0
Total	3083	14	0.5 %	76	2.4 %	57	1.8 %	56	1.8 %

The symbol ‘-’ characterizes samples for which a PCR was not carried out (for salivary glands and abdomens) because the thoraxes tested negative for both *Plasmodium* and *Haemoproteus* spp. for those particular mosquito species

Of the 76 thorax positive mosquitoes, 56 were also positive from the corresponding abdominal extracts (73.6 %). Interestingly, we had difficulty obtaining clean sequences from 15 abdomens of the 56 total that tested positive for *Plasmodium* (multiple peaks were observed throughout the chromatogram indicating the potential for an infection of multiple *Plasmodium* lineages from a bloodmeal obtained from a bird with a co-infection); however, we had no difficulty obtaining clean sequences from thoraxes and salivary glands. Moreover, there were a total of 10 individuals (seven *Cx. stigmatosoma* and three *Cx. tarsalis*) in which at least one body part was infected with a different lineage from the rest of the body (e.g., salivary gland was infected with lineage SPTO_CA_ELW_6P and the abdomen was infected with lineage HOWR_CA_ELW_10P). Lastly, clean sequences for *Haemoproteus* were obtained only from the thoraxes, and not from the abdomens.

### Phylogenetic analysis of mosquito and avian Plasmodium lineages

A phylogenetic analysis based on the cyt *b* gene sequences from both avian and mosquito *Plasmodium* DNA from China Creek Park, along with 16 other *Plasmodium* sequences and 2 *Haemoproteus* sequences obtained from GenBank was performed (Fig. 1). We relied on a combination of GenBank matches, phylogenetic placement, and/or morphological identifications from avian blood smears with a 100 % sequence match to identify as many of the lineages to species level as possible.

**Fig. 4:**
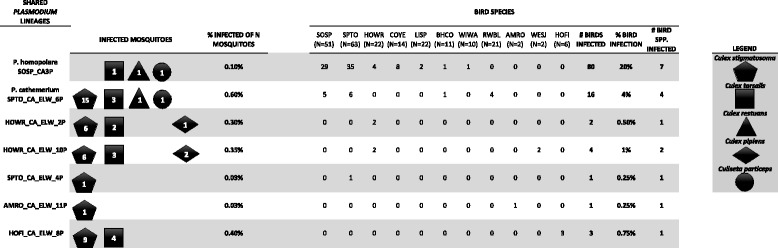
Mosquito and avian species that tested positive for the shared seven *Plasmodium* lineages. Numbers of *Plasmodium-*positive thoraxes are indicated within shapes corresponding to that mosquito species. The legend to the right of the table provides the mosquito species represented for each shape. For the avian species we provide the 4 letter common name code, where SOSP is a Song sparrow (*Melospiza melodia*), SPTO is a Spotted towhee (*Pipilo maculatus*), HOWR is a House wren (*Troglodytes aedon*), COYE is a Common yellowthroat (*Geothlypis trichas*), LISP is a Lincoln’s sparrow (*Melospiza lincolnii*), BHCO is a Brown headed cowbird (*Molothrus ater*), WIWA is a Wilson’s warbler (*Cardellina pusilla*), RWBL is a Red-winged blackbird (*Agelaius phoeniceus*), AMRO is an American robin (*Turdus migratorius*), WESJ is a Western scrub-jay (*Aphelocoma californica*), and lastly, HOFI is a House finch (*Haemorhous mexicanus*)

Based on a 100 % cyt *b* sequence match, 7 out of the 11 *Plasmodium* lineages detected from mosquitoes were also found in birds at China Creek Park. The number of times these 7 overlapping lineages were detected from mosquito thoraxes and bird species is provided in Fig. 4. Lineage SPTO_CA_ELW_6P was the most common lineage found in mosquitoes (20 out of the 76 total, of which 15 were *Cx. stigmatosoma*) and was the second most common lineage in birds “Walther et al., unpublished observations.” Lineage SPTO_CA_ELW_6P had a 100 % match to accession number AY377128 deposited in Genbank that identified the sequence as *Plasmodium cathemerium.* The placement of this lineage in the clade with a known *P. cathemerium* sequence with a posterior probability of 0.99 (Fig. 1) corroborated its identity*.*

Lineage SOSP_CA3P is of particular interest, for it was the predominant lineage found in birds “Walther et al., unpublished observations,” but was rarely found in mosquitoes with only one detected from each of *Cx. tarsalis, Cx. restuans,* and *Cs. particeps*. Lineage SOSP_CA3P has been identified and newly described by Walther et al. [4] as *P. homopolare.*

## Discussion

### ***Plasmodium*** spp. diversity

China Creek Park was selected as our field site due to its high richness of riparian songbird and mosquito species compared to other sites in California. Due to the close proximity of the Park to residences, larvicide based mosquito control was performed almost every week to suppress mosquito populations during the breeding season. Despite mosquito control, 3083 mosquitoes were collected in a two year span, from which 11 *Plasmodium* lineages were detected within the 1.2 km^2^ park. The number of lineages detected from China Creek Park mosquitoes was higher than the ones detected in mosquitoes by PCR in the temperate climates of southwest Pacific islands (11 vs. 4 in Ishtiaq et al. [11]) and in tropical climatic Panama (11 vs. 9 in Loaiza and Miller [21]), and 10 lineages fewer than that detected from mosquitoes collected from 16 locations in tropical forested areas of southern Cameroon [19]. China Creek Park falls within the Mediterranean climatic zone being located in the central valley of California, but due to its location in the Kings River flood plain, it is moister than most parts of the central valley.

Concomitant avian blood samples from birds allowed some sequence comparisons and morphological examinations of blood smears of overlapping bird and mosquito lineages. Lineage SOSP_CA3P was recently described by Walther et al. [4] as a new species: *P. homopolare.* This species was the most common generalist avian parasite in resident (non-migratory) and hatch-year (HY) birds in China Creek Park; in fact, it exceeded bird prevalence of all the other six mosquito-bird shared *Plasmodium* lineages combined (80 birds infected with *P. homopolare* vs. 27 birds infected with the remaining six shared lineages) by almost three times (Fig. 4). However, *P. homopolare* was detected from the thorax of only three mosquitoes representing three different species. The second most common bird and most common mosquito *Plasmodium* lineage detected was lineage SPTO_CA_ELW_6P. It was confirmed to be *P. cathemerium,* which is a well-studied parasite that has a worldwide distribution, and is considered a generalist avian parasite [2].

The remaining five overlapping lineages will be recorded as *Plasmodium* spp. until further careful morphological examination of these parasites have been completed, although two of them, namely lineages HOFI_CA_ELW_8P and Cx.tars_CA_JSC_14P may later be confirmed to be either morphospecies or strains of *P. cathemerium*. Additionally, morphological examinations of lineage HOWR_CA_ELW_10P and comparisons with sequences in GenBank from previous California bird species seem to indicate that this is a new species “Carlson et al., unpublished observations,” and a formal description is forthcoming. The four lineages detected only from mosquitoes are defined only as lineages at present.

Although *Haemoproteus* spp. were detected in our mosquitoes, we don’t consider this an important finding. Abortive sporogonic development takes place in mosquitoes as proven by experimental infections of mosquitos by Valkiūnas et al. [23], and none of the salivary glands in our studies were *Haemoproteus* positive. However, it is interesting that mosquitoes species like *Ae. vexans, An. freeborni, An. punctipennis*, and *Cs. particeps* were positive for *Haemoproteus* spp., indicating that although not considered to be ornithophillic species, they must have taken a blood meal from an infected bird.

### Identification of major and minor potential competent vectors

Vector competence is defined as the ability of an arthropod vector to transmit a pathogen to a new host [43]. Valkiūnas et al. [23] stated that it is important to include experimental research and microscopic approaches in conjunction with molecular methods to detect haemosporodians in vector studies. This is especially true when considering that even when sporozoites are present in salivary glands, they still may not be transmitted [23].

At China Creek Park, limited exposure to avian parasites would be expected in the three *Aedes,* the two *Anopheles* species and all *Culiseta* species because they feed almost exclusively on large and small mammals and very rarely on birds [34]. *Cx. stigmatosoma* and *Cx. thriambus* have strong tendencies to feed on birds [34], although they will feed on frogs and reptiles when given the opportunity “Cornel, unpublished observations.” *Cx. pipiens* complex*, Cx. tarsalis, Cx. restuans,* and *Cx. erythrothorax* are all opportunistic bird, human and large mammal blood feeders with tendencies to feed more on birds, especially fledglings [34].

No *Plasmodium* spp. were detected in the bodies of six mosquito species that do not bite birds frequently (Tables 1, 2, and 3). This was not unexpected, but an interesting exception to this was *Cx. erythrothorax* (297 tested), which appears to be an ornithophillic species at China Creek Park, where an individual mosquito was observed feeding on a bird as we were placing the bird in the Ehrenberg trap in 2011 (Table 1). No conclusions can be made about the role of *Cx. thriambus* because too few were sampled. Two *Cs. particeps* thoraxes were *Plasmodium* positive, but the contiguous salivary glands were negative, indicating a possible minor role of this mosquito in avian malaria transmission. A species in the same genus, *Cs. morsitans*, is considered an important vector of avian *Plasmodium* in eastern North America [44].

We had expected to observe higher *Plasmodium* infection and infectivity in Cx*. pipiens* complex members, considering that in other locations it is implicated to be a major competent vector for avian malaria [20, 45]. Only three infected individuals of 693 collected were positive, and all three were *Cx. pipiens-quinquefaciatus* hybrids. Reeves et al. [46] also reported low prevalence in *Cx. quinquefasciatus* in Kern County, California (just south of our study site), where only 14 out of 711 sampled were positive. Genetic factors can influence susceptibility to infections of vectors of dengue [47] and human *Plasmodium* [48]. Perhaps *Plasmodium* refractory genotypes of *Cx. pipiens* complex members occur in California, which could explain this anomaly.

Three of the 15 mosquito species captured, namely, *Cx. stigmatosoma, Cx. tarsalis,* and *Cx. restuans,* can be referred to, with some degree of confidence, as vectors of *Plasmodium* in China Creek Park, because they had multiple positive salivary gland infections. Similar findings of the role of these mosquito species were reported by Reeves et al. [46], where *Cx. stigmatosoma* had the highest prevalence, followed by *Cx. tarsalis.*

### Vector-parasite-host associations

The most commonly detected *Plasmodium* species in mosquitoes was *P. cathemerium* (Fig. 1), or lineage SPTO_CA_ELW_6P*. P. cathemerium*, which was predominately found in Song sparrows (*Melospiza melodia*) and Spotted towhees (*Pipilo maculatus*) “Walther et al., unpublished observations,” was mainly detected in *Cx. stigmatosoma,* followed by *Cx. tarsalis,* and one detection from *Cx. restuans.* Lineage HOFI_CA_ELW_8P, which was only found in House finches (*Haemorhous mexicanus*) “Walther et al., unpublished observations,” was also predominantly found in *Cx. stigmatosoma* followed by *Cx. tarsalis*. Lineage Cx.tars_CA_JSC_14P, not found in birds, was only detected in one *Cx. tarsalis*.

Attention should be drawn to the extreme difference in detection rates of *P. homopolare* (lineage SOSP_CA3P) in the avian hosts and the mosquitoes. *P. homopolare* was detected in 83 of 399 birds (20 %) sampled at China Creek Park, which primarily infected resident birds, of which 4 individuals were HY birds, and also included 6 individuals representing 3 migratory species [4]. However, *P. homopolare* was only detected in 3 of 76 *Plasmodium* infected mosquitoes. Interestingly, no *Cx. stigmatosoma* were infected with *P. homopolare*, despite the fact that *Cx. stigmatosoma* had the highest infection/infectivity rates for all other *Plasmodium* species and lineages out of all the other mosquito species*. Cx. stigmatosoma,* therefore, appears to be a general vector of several *Plasmodium* species, except for *P. homopolare*, but more sampling is needed to confirm this*.* Of all the birds infected with *Plasmodium* at China Creek Park*,* 68 % were infected with *P. homopolare* [4]*,* yet there were far fewer detections of this parasite in mosquitoes than other lineages that were much less common in birds. We are confident that local transmission of *P. homopolare* is taking place at China Creek Park due to infections in resident HY birds. This disparity may be attributed to a combination of our sampling scheme and refractoriness of some mosquito species to infections of certain *Plasmodium* species.

### Effects of sampling bias

Multiple mosquito collecting methods were used to maximize mosquito diversity, but also to collect mosquitoes at different physiological states and ages. EVS and bird-baited traps collect a variety of mosquito species, depending on the season, these mostly are unfed nulliparous females (>65 %) [49–51]. Hence, the majority of the mosquitoes collected in these traps have never come into contact with *Plasmodium* infected birds. Gravid traps, on the other hand, collected older mosquitoes that have taken and digested at least one blood meal, and therefore had potential exposure to *Plasmodium* infected hosts, which enhances chances of collecting infected mosquitoes. However, gravid traps collected a lower diversity of mosquito species overall [52].

Higher prevalence in *Cx. stigmatosoma,* which were mainly collected in gravid traps, may be a reflection of the fact that we sampled the previously blood fed proportion of this population. Perhaps, high infection/infectivity rates would also have been obtained in *Cx. tarsalis* had we collected similar numbers of this species in gravid traps. *Cx. tarsalis* were collected in EVS and Ehrenberg traps, which likely led to sampling mostly first time blood feeders. This notion was also supported by the results from the GLM analysis on the prevalence of *Plasmodium.* Gravid traps resulted in only a slightly higher *Plasmodium* prevalence compared to EVS traps after adjustment for differences among species. *Plasmodium* prevalence in *Cx. tarsalis* was not significantly different from that in *Cx. stigmatosoma* after adjustment for trap type, suggesting that if *Cx. stigmatosoma* and *Cx. tarsalis* could be collected equally in both trap types, they would probably have very similar *Plasmodium* prevalence.

Hence, because of a trap bias, we are unable to confirm whether *Cx. tarsalis* or Cx. *stigmatosoma* play a more dominant role in *Plasmodium* transmission. If we had used only EVS traps then we would not have discovered the major role of *Cx. stigmatosoma* as a vector. Gravid traps were more efficient than other traps at collecting *Cx. pipiens* complex and *Cx. restuans*, both of which had much lower infection/infectivity rates than *Cx. stigmatosoma*. Consequently, we can conclude that *Cx. stigmatosoma* is a much more significant avian *Plasmodium* vector than *Cx. pipiens* complex and *Cx. restuans* at China Creek Park*.* This emphasizes the importance of using a multitude of trapping techniques that will address the specific biology of multiple mosquito species.

### Fidelity in vector competence of some Plasmodium species

Absence of *P. homopolare* in *Cx. stigmatosoma* and low or absent *Plasmodium* prevalence in other ornithophilic species, such as *Cx. erythrothorax, Cx. pipiens* complex, and *Cx. restuans* suggests that not all *Culex* species are equally capable of transmitting all species of *Plasmodium*. Positive salivary glands from only three out of the five mosquito species that had *Plasmodium* positive thorax and/or abdomens reduces the number of mosquito species that we can conclude were involved in avian malaria transmission. Had we relied solely on whole body extraction results, we would have over-estimated the number of mosquito species involved in avian malaria transmission. Some species of mosquitoes were infected with multiple *Plasmodium* species/lineages, which could easily suggest that *Culex* mosquitoes are generalist *Plasmodium* vectors. However, because of the attention given to mosquito sampling and dissection of salivary glands, our study suggests that there is some fidelity in avian *Plasmodium*-*Culex* associations*,* which is contrary to the general held view that *Culex* are generalist avian malaria vectors [12]. Follow up vector competence studies would be warranted to provide corroborative support that specific avian *Plasmodium*-*Culex* associations appear to exist, as our field study results suggest. Inability of, for example, *Cx. pipiens* complex and *Cx. erythothorax* in vector competence trials to transmit any of the 11 *Plasmodium* lineages from China Creek Park would explain the lack of *Plasmodium* detection from these species at China Creek Park. If these species are capable of transmitting *Plasmodium* in vector competence trials, then sampling error has to be considered to explain our observations. Similarly, to remove doubt based on sampling phenomena, it would be especially relevant to also comparatively infect *Cx. stigmatosoma* and *Cx. tarsalis* with *P. homopolare* to determine their ability to transmit this parasite.

The heterogeneities in the transmission of avian malaria by mosquitoes reported in this study does provide some support for the hypothesis that vector mosquito species can influence the structuring of avian malaria parasites in host populations by acting as a compatibility filter for specific parasite species at a given site. China Creek Park has a high level of mosquito species richness, which would cause a so-called “dilution effect” in the importance of the contribution of each competent mosquito species in shaping the parasite community structure. Each species would have their own compatibility filter for specific *Plasmodium* spp., but collectively they would allow for a high level of parasite diversity within the host population, especially considering that China Creek Park also has a high level of migratory bird species richness. However, if, for example, a site that has a lower mosquito species richness, but has an equivalent level of resident host species richness as China Creek Park and is exposed to the same migratory species (thus, having equal opportunity to the same parasite species exposure), then the effect of a compatibility filter would be more evident. The structuring of the parasite community within the host population would be less diverse. Although making conclusions on the role of mosquitoes in shaping the community structure in a host population based on the reporting of only one site in this study is speculative, we have collected data from a second site in California in the summer of 2014 that would help support this hypothesis, but will be reported at a later date “Carlson et al., unpublished observations.” Nonetheless, it does highlight the need to investigate the role of mosquitoes to fully understand the epidemiology of avian malaria, and especially how that role changes in different ecosystems.

### The unknown effects of Plasmodium co-infections in the vector

It has been previously reported that the primers used by many of the current avian malaria studies tend to preferentially bind to one cyt *b* gene sequence of one parasite over others in a case of a co-infection [53, 54]. The mechanism for which this happens is poorly understood and seems to be independent of the abundance of each parasite within a blood and/or tissue sample. This could cause problems when pools or whole bodies are used for screening of infections. By doing so, primers that are selective for one particular species might selectively amplify only one parasite in the presence of a co-infection, while the other parasites may never be detected. When identifying potential competent vectors, it would be advisable to avoid pooling samples until the development of markers that can differentiate between species are developed.

In this study, we detected co-infections in individual mosquitoes. Fifteen abdomens of the 56 that tested positive for *Plasmodium* revealed co-infections due to the presence of multiple peaks throughout the chromatogram. Of the remaining infected mosquitoes in which we were able to get a clean sequence from the abdomens, there were 10 individuals (7 *Cx. stigmatosoma* and 3 Cx*. tarsalis*) in which at least one body part was infected with a different lineage from the rest of the body. This elucidates how the role of mosquitoes in the transmission of avian malaria parasites is most likely severely underestimated with the current molecular markers. The mechanisms behind species-specific parasite interactions with one another in the small space of a mosquito vector still remains an unexplored area in this field.

## Conclusions

The use of diverse mosquito trapping methods and identification of *Plasmodium* infection and infective status from individual mosquito abdomens, thoraxes, and salivary glands, allowed us to more precisely identify the role of mosquitoes in avian malaria transmission in China Creek Park. If we had relied solely on EVS-CO_2_ baited traps that attract and collect host-seeking mosquitoes, we would have missed the important role of *Cx. stigmatosoma* because this mosquito was mostly collected in gravid traps. Calculating a minimum prevalence for each mosquito species based on pools of whole-bodied mosquitoes likely would have led to the conclusion that *Cx. tarsalis* was the only vector of avian malaria in China Creek Park. Low *Plasmodium* detections and lack of salivary gland positives suggest a minor role for *Cs. particeps* and *Cx. pipiens* complex. Confidence in our hypothesis that *Cx. pipiens* complex plays a very limited role in transmission also would have been weak had we not used gravid traps. The efficiency of gravid traps over EVS traps produced a much higher sample size of *Cx. pipiens* complex, reducing the likelihood of sampling bias errors.

A high diversity of *Plasmodium* species are present in central California, but is dominated by a few species such as *P. homopolare* and *P. cathemerium* in the avian population*.* Two species of *Culex* mosquitoes, namely *Cx. stigmatosoma* and *Cx. tarsalis,* appear to be the primary vectors of avian *Plasmodium.* However, *Cx. stigmatosoma* does not appear to be a vector of *P. homopolare* based on our trap data, but this should be followed up with vector competence assays to prove this*.* Further field sampling and laboratory experimental infections with multiple mosquito species will be required to determine the vector(s) of *P. homopolare.* Two other common ornithophilic *Culex* mosquitoes, *Cx. erythrothorax* and *Cx. pipiens* complex, are either incapable or highly inefficient vectors of avian malaria in central California. This leads us to conclude that some degree of specialized associations of *Plasmodium* species and mosquitoes occur in California.

## Abbreviations

CA: Correspondence analysis
Cyt b: Cytochrome oxidase subunit-*b*
EVS: Encephalitis virus surveillance
HY: Hatch-year
PCR: Polymerase chain reaction
